# Intravascular Papillary Endothelial Hyperplasia (Masson’s Tumor) of the Thumb Presenting as a Giant Cell Tumor of Tendon Sheath: A Case Report and Literature Review

**DOI:** 10.7759/cureus.91817

**Published:** 2025-09-08

**Authors:** Grigorios Kastanis, Constantinos Chaniotakis, Konstantinos Zampetakis, Nikolaos Ritzakis, Ioannis Ktistakis

**Affiliations:** 1 Hand Reconstruction Unit, Venizeleio General Hospital, Heraklion, GRC; 2 Orthopaedic Department, Venizeleio General Hospital, Heraklion, GRC

**Keywords:** giant cell tumors, hand mass, hemangioma, intravascular papillary endothelial hyperplasia (masson's tumor), thumb lesion

## Abstract

Masson's tumor, also known as intravascular papillary endothelial hyperplasia (IPEH), is a rare benign vascular lesion of the skin and subcutaneous tissues. It represents the organization and recanalization of a thrombus within a normal vessel, vascular malformation, or hematoma. Although it can occur in any part of the body, its presence in the hand can be particularly challenging to differentiate from other soft-tissue lesions. We present a case of Masson's tumor located in the distal phalanx of the thumb. Initial magnetic resonance imaging (MRI) examination suggested a giant cell tumor of the tendon sheath; however, following surgical excision, histopathological evaluation confirmed the diagnosis of Masson’s tumor. This case highlights the critical importance of histopathological examination, as Masson’s tumor can closely mimic other soft-tissue lesions.

## Introduction

Masson first described this lesion in 1923, originating from an ulcerated hemorrhoidal vein, and named it “hémangioendothéliome végétant intravasculaire.” He considered the lesion to be a specific type of hemangioma, which in its late stages resembled an organized thrombus [1]. In 1932, Henschen proposed that this lesion was a reactive process rather than a neoplastic one [2,3]. Later, in 1976, Clearkin and Enzinger described how intraluminal thrombosis leads to papillary endothelial hyperplasia and introduced the term intravascular papillary endothelial hyperplasia (IPEH) [4,5]. IPEH has been reported in the hand, forearm, neck, oral mucosa, salivary glands, and in both arteries and veins [6]. When it occurs in the hand, it is particularly challenging to differentiate from other soft-tissue lesions based solely on clinical or magnetic resonance imaging (MRI) diagnosis [7].

IPEH can mimic several other soft-tissue tumors, particularly angiosarcoma, making accurate diagnosis essential to avoid unnecessary aggressive treatment [8]. IPEH, as a benign vascular lesion, does not metastasize, and its surgical excision is associated with an excellent prognosis. In contrast, angiosarcoma, as a malignant vascular tumor, is aggressive, with a high recurrence rate and substantial propensity for metastatic dissemination. Management requires wide excision, often followed by radiotherapy and/or chemotherapy, and the overall prognosis remains poor [9]. The purpose of this study is to emphasize the importance of histopathological examination in the diagnosis of IPEH. While the prognosis of IPEH is excellent, its clinical and radiological similarity to malignant lesions-such as angiosarcoma-makes differentiation difficult without histological confirmation.

## Case presentation

**Figure 1:**
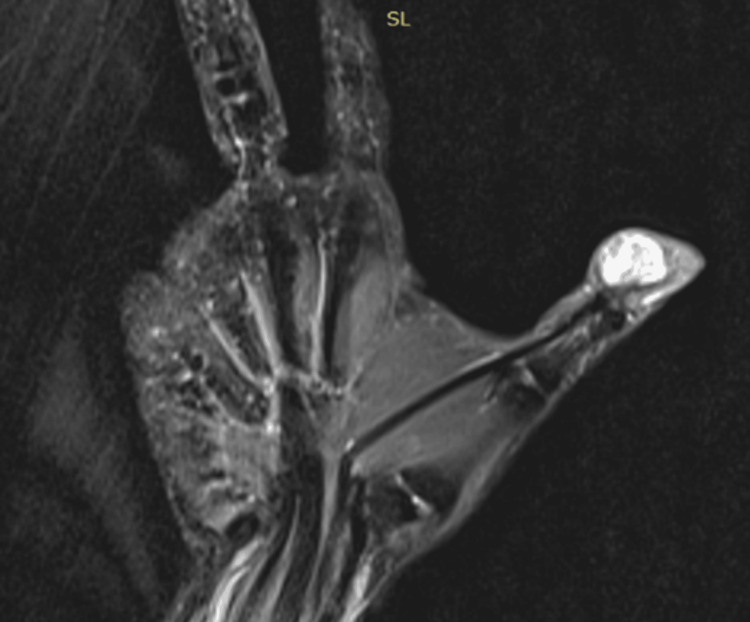
T2-weighted MRI depicts the soft-tissue mass on the palmar surface of the thumb MRI: magnetic resonance imaging

A 46-year-old woman presented with a slowly growing mass on the volar distal phalanx of the right thumb. The patient reported that the mass had been gradually increasing in size over the past two years, without any history of trauma. She also noted the limited functional ability of the thumb. Physical examination revealed a 3 × 2 cm, round, non-tender lesion on the volar side of the distal interphalangeal (DIP) joint of the thumb. The mass was soft in consistency, with normal overlying skin. Neurovascular status was intact, and there were no signs of infection. Laboratory inflammatory markers were within normal limits. MRI demonstrated a subcutaneous, lobulated, homogeneous lesion originating near the flexor pollicis longus tendon, measuring 12 × 16 × 12 mm. The radiological diagnosis was consistent with a giant cell tumor of the tendon sheath (GCTTS) (Figure 1).

**Figure 2:**
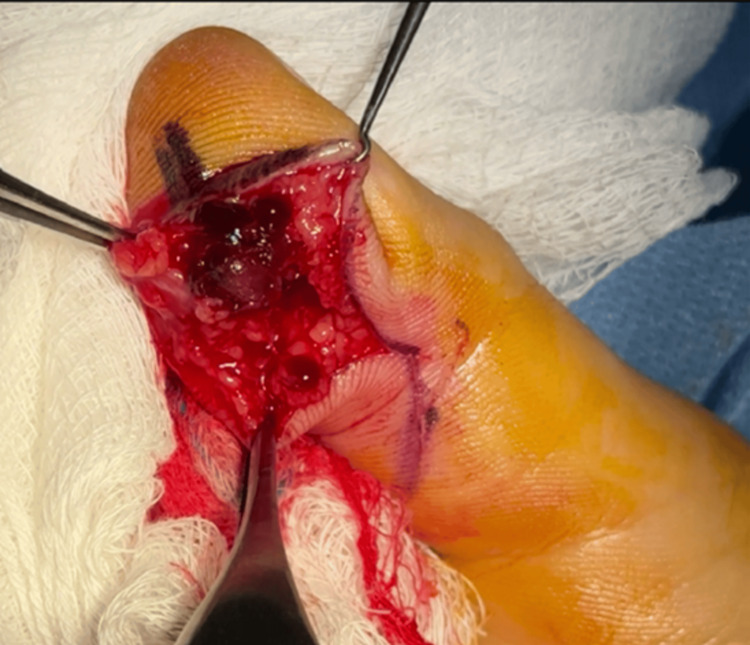
The intraoperative view revealed thrombus formation in the center of the mass

Due to the patient's desire for removal, a surgical excisional biopsy was planned. Under the WALANT (Wide Awake Local Anesthesia No Tourniquet) technique, a Bruner zigzag incision was made over the volar aspect of the DIP joint. Intraoperatively, a well-circumscribed, brown-black, rubbery lesion was identified, with adhesions to the surrounding soft tissues and compression of the tendon sheath. Thrombus formation was noted in the center of the mass (Figure 2).

**Figure 3:**
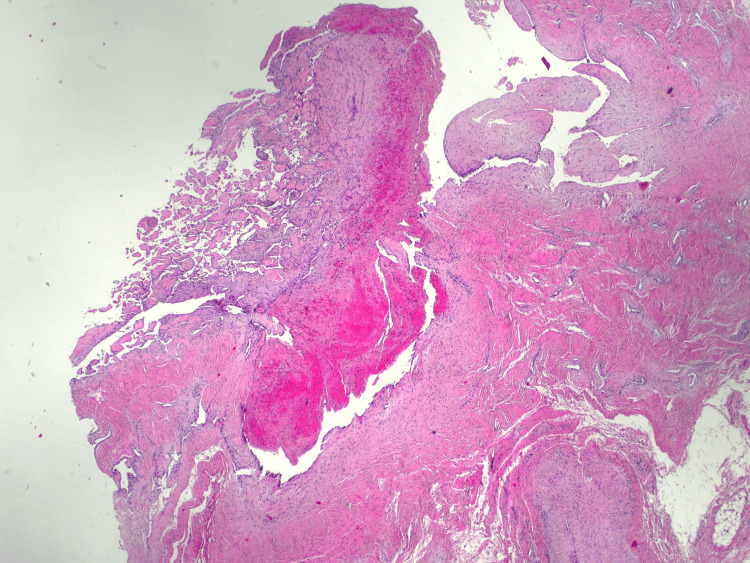
Microscopically, dilated vascular spaces, reminiscent of a hemangioma, were recognized, with the presence of thrombus and papillary endothelial hyperplasia; papillary structures, within a fibrous connective stroma, which are lined by a single layer of plump endothelial cells, inside the lumina of a dilated vessel

The specimen was sent for histopathological examination. Microscopic analysis revealed dilated vascular spaces with intravascular thrombus and papillary endothelial hyperplasia-findings consistent with IPEH, also known as Masson’s hemangioma (Figure 3).

There were no intraoperative or postoperative complications. The patient achieved excellent functional recovery, with no clinical signs of recurrence during a one-year follow-up, and therefore, no further imaging evaluation was performed.

## Discussion

IPEH accounts for approximately 2%-4% of benign and malignant vascular tumors of the skin and subcutaneous tissues [7]. It occurs at a similar rate in both sexes, although some studies suggest a higher incidence in women than in men, with a female-to-male ratio of 1.2:1.0 and an average age of presentation of 34 years [10]. IPEH can occur in any part of the body, with reported localizations including the oral mucosa, lip, thyroid, maxillary sinus, parotid gland, lung, superior vena cava, adrenal gland, renal vein, forearm, foot, intracranial region, and hand [11].

The etiopathogenesis of IPEH remains unclear. In many cases, a preceding trauma is considered the most common contributing factor; however, idiopathic cases have also been reported. Trauma may result in hematoma formation and thrombosis, after which circulating macrophages release growth factors that stimulate endothelial proliferation [12]. Kitagawa et al. suggested that this cellular proliferation may be influenced by hormonal factors or growth factors such as basic fibroblast growth factor (bFGF) [13].

Three types of IPEH have been described in the literature, along with their relative incidences [14]: Type I (pure or primary form) represents 56% of cases and arises de novo within dilated blood vessels, most commonly veins, and only rarely in arteries. Type II (mixed or secondary form) comprises 40% of cases and originates within preexisting vascular lesions such as varices, aneurysms, hemangiomas, arteriovenous malformations (AVMs), lymphangiomas, or pyogenic granulomas. Type III (extravascular form) is the rarest form (4%), which arises within a hematoma. Our case had no reported history of trauma and is therefore categorized as Type I.

When Masson’s tumor appears in the hand, it presents a significant diagnostic challenge, as it can be confused with other vascular neoplasms such as angiosarcoma, hemangioendothelioma, hemangioma, and Kaposi sarcoma [14]. On Doppler ultrasound, it typically appears as a hypervascular lesion, while MRI usually reveals an enhancing homogeneous mass [4,6]. However, these imaging findings are non-specific and offer limited preoperative diagnostic value [6]. A definitive diagnosis of IPEH can be made through histological examination and immunohistochemical staining [4,7,11,12,14]. Histopathology helps differentiate IPEH from angiosarcoma, which may appear microscopically similar but almost never arises within the lumen of a vessel. Accurate diagnosis prevents unnecessary surgical procedures and avoids the use of radiation therapy [7]. In our case, the initial MRI suggested a GCTTS, but the final diagnosis of IPEH was made through histopathological evaluation.

Treatment of Masson’s tumor consists of conservative surgical excision, which generally has an excellent prognosis-except in rare cases of intracranial involvement, which can compromise the patient’s neurological status, may recur, and have been reported to be fatal [7,13]. One of the challenges in treatment is the lack of consensus regarding surgical margin size; however, there is no evidence in the literature supporting the need for wide excision margins [7,12-14].

Recurrence has been reported in approximately 15% of cases, typically associated with incomplete excision or the presence of an underlying co-existing vascular lesion [15]. Although postoperative management guidelines are lacking, radiotherapy and chemotherapy have been reported in cases of recurrence-but without well-defined indications [12,15].

## Conclusions

Masson’s tumor is a benign vascular lesion that may grow either rapidly or slowly and tends to occur in the upper extremities. It often mimics other vascular or skin tumors, and only histopathological examination can reliably distinguish it from other benign and malignant lesions. Complete surgical excision is the gold standard treatment, and the prognosis for IPEH is generally excellent.
